# A Fructan 6-Exohydrolase from *Orobanche cumana* Boosts Waterlogging Tolerance in Parasitic and Root Tissues

**DOI:** 10.3390/plants15091326

**Published:** 2026-04-27

**Authors:** Rui Xu, Yannan Li, Lele Li, Ruixuan Zhao, Runyao Bai, Hada Wuriyanghan, Yanyan Fan

**Affiliations:** 1 School of Life Science, Inner Mongolia University, Hohhot 010030, China; 15071201086@163.com (R.X.); lyn15848497757@163.com (Y.L.); ll15960071522@163.com (L.L.); ruixuanzhao972@163.com (R.Z.); bairunyaowangyi@163.com (R.B.); 2 Key Laboratory of Herbage & Endemic Crop Biology, Ministry of Education (IMU), Hohhot 010030, China; 3 Department of Medicine, Ordos Institute of Technology, Ordos 017000, China

**Keywords:** sunflower, *Orobanche cumana*, fructan, 6-exohydrolase, waterlogging tolerance, IAA

## Abstract

Plants adapt to abiotic stresses by a variety of physiological and molecular mechanisms, among which the root plays important roles via responding to underground and soilborne signals. Fructan is a polysaccharide involved in energy metabolism and stress adaptation. *Orobanche cumana* is a holo-parasitic plant that mainly attaches to the root of the host sunflower (*Helianthus annuus*). Oc6-FEH, a fructan 6-exohydrolase from *O. cumana*, is involved in both fructan metabolism and flooding responses. Expression of *Oc6-FEH* is induced by flooding and indole-3-acetic acid (IAA). Oc6-FEH possesses fructan catabolism activity and is associated with fructose release. Overexpression of *Oc6-FEH* in the host sunflower reduces malondialdehyde (MDA) and hydrogen peroxide (H_2_O_2_) accumulation, boosts the activities of antioxidant enzymes, including peroxidase (POD) and superoxide dismutase (SOD), and enhances photosynthetic performance. The expression level of *Oc6-FEH* was found to be positively associated with the flooding tolerance of invading *O. cumana*, which is connected to the host root. Furthermore, IAA treatment also improved the flooding tolerance of *O. cumana*. In summary, the metabolism of fructan and the activity of Oc6-FEH were demonstrated to ameliorate waterlogging stress. Oc6-FEH provides a promising genetic target for the improvement of flooding tolerance in crops.

## 1. Introduction

Flooding is a major abiotic stress that constrains global agricultural production and natural vegetation distribution [1,2,3]. With climate change intensification and extreme rainfall events becoming more frequent, the severity and impact of flooding are increasing, posing a serious threat to plant growth, crop yield, and ecosystem stability [2,4]. Flooding rapidly reduces gas diffusion and depletes soil oxygen (O_2_) within hours [5]. Plants depend on soil oxygen for aerobic respiration and energy metabolism; hypoxia directly inhibits the mitochondrial electron transport chain, impairing adenosine triphosphate (ATP) synthesis [6,7]. Furthermore, hypoxic waterlogged soils lead to the accumulation of toxic ions, such as Fe^2+^ and Mn^2+^, and typically cause a shift in pH towards neutrality, which can exacerbate stress on root systems [8,9].

The detrimental effects of flooding on plants include morphological and growth perturbations, including inhibited root elongation and root hair development as well as reduced root biomass, as well as aerial symptoms, including leaf chlorosis, wilting, premature abscission, stunted growth, and reduced biomass [10]. Severe or protracted waterlogging can ultimately cause plant death, primarily through root rot and a complete breakdown of photosynthetic function [8,10]. Waterlogging severely impairs the root’s capacity for water and nutrient uptake, which compromises water transport, lowering the leaf water potential and triggering stomatal closure, which in turn causes drastic declines in transpiration and photosynthetic rates [11,12]. The photosynthetic apparatus is further damaged by chlorophyll degradation, a drop in photosystem II efficiency, and the inhibition of a key enzyme, ribulose-1,5-bisphosphate carboxylase/oxygenase, collectively hindering the production of photoassimilates [13].

Plants adapt to waterlogging through the integrated actions of ethylene and other hormones [14]. Ethylene rapidly builds up in flooded tissues and activates downstream ethylene response factors (ERFs) to orchestrate the expression of adaptive genes [7]. To adapt to waterlogged soils, plants remodel their root system architecture (RSA) through the coordinated action of auxin and ethylene. Ethylene modulates both auxin biosynthesis and polar transport, thereby reshaping the auxin gradient in the root tip. This hormonal interplay suppresses primary root elongation while stimulating the formation of adventitious roots [15,16]. Adventitious roots, which emerge from the stem or hypocotyl base, facilitate internal aeration and alleviate hypoxia in the root zone [9,10]. Consistently, exogenous application of IAA promotes adventitious rooting and enhances waterlogging tolerance [17]. 

Under waterlogging stress, mitochondrial aerobic respiration is impaired and electron leakage from the disrupted respiratory chain becomes a major source of ROS. These ROS can damage cellular membranes, oxidize proteins, and damage DNA, aggravating cell death [18,19]. Conversely, ROS also function as signaling molecules in hypoxia perception and response. For example, the NADPH oxidase respiratory burst oxidase homolog (RBOH) converts apoplastic superoxide (O_2_•^−^) into hydrogen peroxide (H_2_O_2_), which subsequently activates calcium (Ca^2+^) influx and the mitogen-activated protein kinase (MAPK) cascade, leading to transcriptional reprogramming [19,20].

Faced with the energy crisis caused by waterlogging, plants reprogram their carbon metabolism, enhancing processes such as starch degradation, glycolysis, and the pyruvate fermentation pathway [21]. In *Oryza sativa*, for instance, hypoxia upregulates the expression of α-amylase genes such as *RAMy3D* to fuel glycolysis [2]. As an important carbon reserve, fructan may be mobilized by hydrolytic enzymes like fructan exohydrolase (FEH) to release fructose for energy production or osmoregulation [22]. The role of the fructan degradation pathway, however, remains poorly characterized in the context of parasitic plant interactions. Fructan serves as a vital storage and stress-response carbohydrate in approximately 15% of higher plants, including sunflower [23], which can synthesize inulin-type fructans [24]. Fructan exohydrolases hydrolyze terminal glycosidic bonds from the non-reducing ends of fructan chains, releasing fructose monomers. Based on their linkage specificity, FEHs are categorized primarily into 1-FEH (hydrolyzing β-(2,1) bonds), 6-FEH (hydrolyzing β-(2,6) bonds), and dual-specificity 6&1-FEH, which acts on both linkage types [25,26,27,28].

The expression and activity of FEHs are strongly induced by various environmental stresses and play multiple roles in plant stress resistance. Under abiotic stresses such as low temperature and drought, FEH mediates degradation of fructan, facilitates the rapid release of soluble sugars, and provides carbon sources and energy [29,30]. Beyond carbon mobilization, fructans themselves may directly stabilize membrane structures by interacting with lipids, thereby enhancing tolerance to freezing and drought [31]. Recent studies have also unveiled novel functions for *FEHs* in biotic stress responses. For example, maize *6-FEH* is induced upon bacterial infection and exhibits substrate specificity for levan, suggesting a role in degrading microbial-derived fructans during plant–microbe interactions [32]. This supports the concept of “sweet immunity”, where carbohydrates, including fructan, and their oligosaccharides can act as signaling molecules to activate the plant’s immune system [33,34].

*Orobanche cumana* is a holo-parasitic plant that specifically attaches to the roots of sunflower [35]. This parasitic weed lacks its own functional root system and relies entirely on a specialized organ, the haustorium, to withdraw water, nutrients, and carbohydrates from the host vascular system [36,37]. Parasitism by *Orobanche* species imposes a significant metabolic burden on the host, acting as a strong sink for photosynthates and competing with host sinks for carbon resources [38]. In infected hosts, the competition for carbon resources between the parasite and host metabolic demands can exacerbate stress-induced carbon starvation, affecting growth and survival [39]. Parasitic infection has been shown to alter host carbohydrate dynamics, including sucrose, starch, and soluble sugar pools [40].

However, in the complex context of parasitic interactions and waterlogging stress, the regulatory mechanism of host fructan metabolism remains unclear. Therefore, this study aimed to investigate whether waterlogging stress induces the expression of *Oc6-FEH* via IAA and other signals and to determine the role of *Oc6-FEH* in modulating the waterlogging tolerance of the parasitic system.

## 2. Results

### 2.1. Expression Analysis of Oc6-FEH in Response to Waterlogging and Fructan Treatment

**Figure 1:**
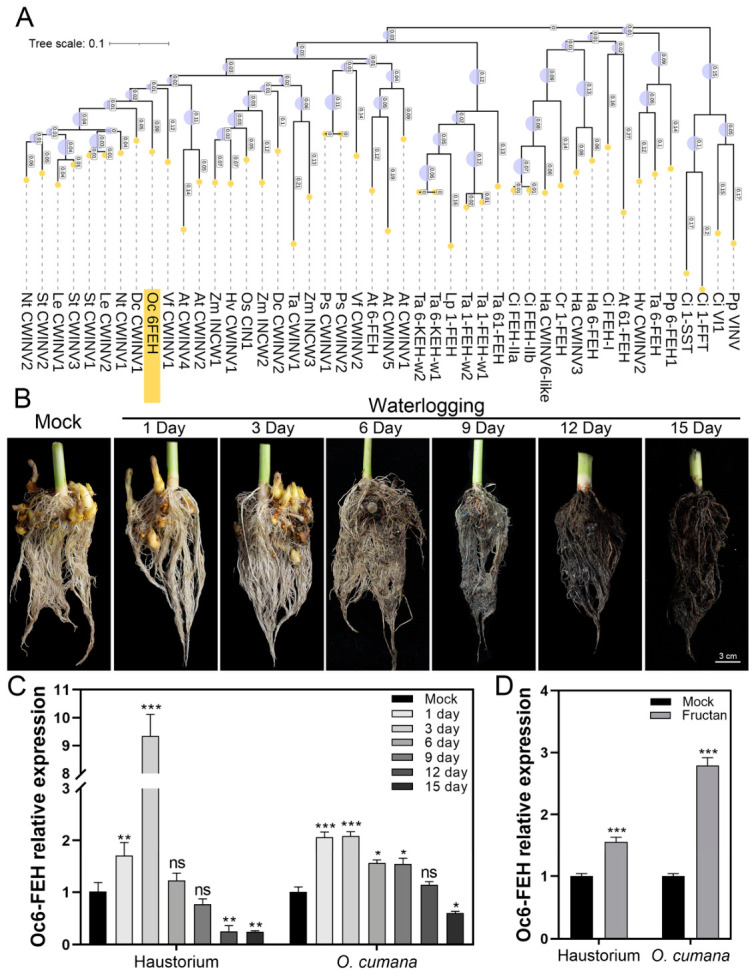
Phylogenetic tree and expression pattern analysis of *Oc6-FEH* from *O. cumana*. (**A**) Phylogenetic tree of fructan-metabolism-related enzymes, including Oc6-FEH. Species abbreviations are as follows: At, *Arabidopsis thaliana*; Bv, *Beta vulgaris*; Ci, *Cichorium intybus*; Cr, *Campanula rapunculoides*; Ta, *Triticum aestivum*; Hv, *Hordeum vulgare*; Lp, *Lolium perenne*; Pp, *Phleum pratense*; Cd, *Cistanche deserticola*; Oc, *Orobanche cumana*. Purple circles of different sizes represent bootstrap values (larger circles indicate higher bootstrap support). (**B**) Phenotypic observation of sunflower roots parasitized by *O. cumana* under waterlogging stress. Bars, 3 cm. (**C**) RT-qPCR analysis of *Oc6-FEH* expression in *O. cumana* stems and haustoria under waterlogging stress. (**D**) *Oc6-FEH* expression in haustoria and *O. cumana* stem tissues in response to exogenous fructan (0.02%) treatment. Data are means ± SD (*n* = 3). Specific statistical tests used: one-way ANOVA (**C**), Student’s *t*-test (**D**). * *p* < 0.05, ** *p* < 0.01, *** *p* < 0.001; ns, not significant.

Here, we aimed to characterize fructan-exohydrolase-encoding genes in *O. cumana*. An *FEH* gene named *Oc6-FEH* was obtained from the *O. cumana* transcriptome sequence by sequence searching and annotation. A phylogenetic tree was constructed by using 6-FEH, cell wall invertase (CWINV/INCW), sucrose:sucrose 1-fructosyltransferase (1-SST), fructan:fructan 1-fructosyltransferase (1-FFT), vacuolar acid invertase (VINV), and fructan 1-exohydrolase (1-FEH) from *Arabidopsis*, sugar beet, chicory, sainfoin, wheat, barley, and other species. As a result, Oc6-FEH clustered with cell wall invertase from both monocots and dicots (Figure 1A), indicating that Oc6-FEH might have evolved from a CWINV/INCW ancestor. The multiple sequence alignment of Oc6-FEH with 6-FEH proteins from *Arabidopsis thaliana* and *Beta vulgaris* is shown in Supplementary Figure S1, which confirms the presence of conserved GH32 domains and key catalytic residues.

To investigate whether waterlogging affects the parasitic system, we first examined the phenotype of sunflower roots parasitized by *O. cumana* under waterlogging stress. *O. cumana* is a root parasite, and we found that overwatering would negatively influence its growth. The host sunflower root appeared to suffer from melanism at 6 days post waterlogging (dpw), and the number of attached *O. cumana* parasites radically reduced at the same time points (Figure 1B). We then quantified *Oc6-FEH* transcript levels in *O. cumana* and haustorium tissues at different time points after waterlogging. *Oc6-FEH* expression was induced by waterlogging in both *O. cumana* and haustorium tissues at different time points. In haustorium, *Oc6-FEH* expression peaked at 3 dpw, with 9-fold that of the mock group, and then gradually declined to the basal level at 9 dpw. In *O. cumana* tissue, *Oc6-FEH* expression doubled that of the mock group and then gradually declined to the basal level at 12 dpw (Figure 1C). Sunflower seedlings were cultivated in the hydroponic system and inoculated with *O. cumana*. When 0.02% fructan was added to the hydroponic system (targeting the root zone where the sunflower roots and *O. cumana* inoculation sites were located), *Oc6-FEH* expression increased by 1.6-fold in haustorium and 2.8-fold in *O. cumana* tissues at 7 days (Figure 1D).

### 2.2. Detection of Oc6-FEH Promoter Responsiveness to Waterlogging, IAA, and MeJA

**Figure 2:**
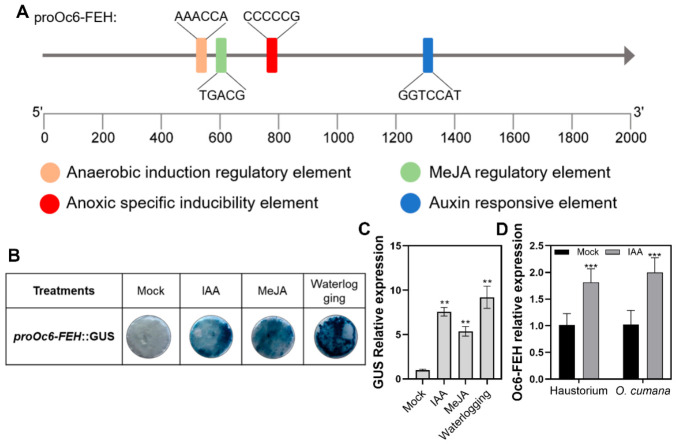
Cloning and functional validation of the *Oc6-FEH* promoter. (**A**) Schematic diagram of predicted cis-acting elements in the *Oc6-FEH* promoter region. The anaerobic induction element, anoxic-specific inducibility element, MeJA-responsive element, and auxin-responsive element are indicated. (**B**) GUS staining of *N. benthamiana* leaves transiently expressing *proOc6-FEH*::GUS after different treatments. The tissues were treated for 24 h with mock, 100 nM IAA, 100 μM MeJA, or waterlogging. (**C**) RT-qPCR analysis of GUS transcript levels in the infiltrated leaf areas shown in (**B**). Data are means ± SD (*n* = 3). (**D**) RT-qPCR analysis of *Oc6-FEH* expression in haustoria and *O. cumana* tissues 12 h after treatment of parasitized sunflower plants with 100 nM IAA. Data are means ± SD (*n* = 3). Specific statistical tests used: one-way ANOVA (**C**), Student’s *t*-test (**D**). ** *p* < 0.01, *** *p* < 0.001; ns, not significant.

To investigate the transcriptional regulation of *Oc6-FEH,* we cloned its promoter region and analyzed the cis-acting elements. The 2000 bp promoter region of *Oc6-FEH* contains multiple putative cis-acting elements predicted to be involved in abiotic stress and phytohormone signaling. Specifically, we identified anaerobic induction elements, anoxic-specific inducible elements, a MeJA-responsive element, and auxin-responsive elements (Figure 2A).

The *proOc6-FEH*::GUS reporter in *N. benthamiana* leaves showed low basal activity, with barely detectable GUS staining in the mock group. In contrast, clear GUS signals were observed following treatments with IAA, MeJA, or waterlogging (Figure 2B), and the RT-qPCR experiment indicated that GUS expression was increased by 9.2, 7.6, and 5.4-fold upon treatment with waterlogging, IAA, and MeJA, respectively (Figure 2C). In sunflower plants parasitized by *O. cumana*, IAA treatment induced *Oc6-FEH* expression in both haustoria and *O. cumana* tissues. RT-qPCR analysis showed that IAA treatment significantly enhanced the expression of *Oc6-FEH* in parasitized tissues, with a 1.8-fold increase in haustorium and a 2.0-fold increase in *O. cumana* tissue (Figure 2D), indicating that, under natural parasitism conditions, *Oc6-FEH* expression is also induced by IAA.

### 2.3. Assessment of Oc6-FEH Exohydrolase and Fructan Catabolism Activity

**Figure 3:**
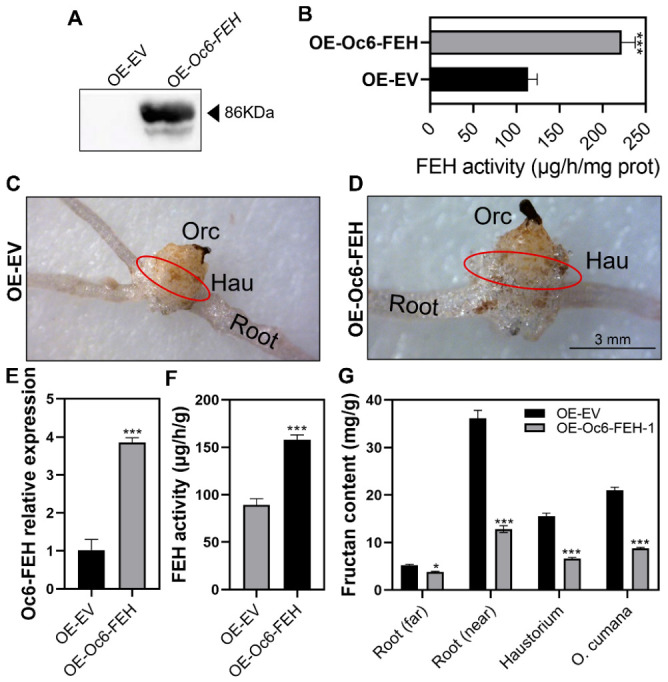
Enzymatic activity of Oc6-FEH and its effect on fructan metabolism in the parasitic system. (**A**) SDS-PAGE analysis of recombinant Oc6-FEH-GST protein expressed in *E. coli*. The arrow indicates the target band at approximately 86 kDa. (**B**) FEH activity assay of bacterial lysates expressing *Oc6-FEH.* Data are means ± SD (*n* = 3). (**C**,**D**) Representative images of sunflower plants transiently overexpressing *Oc6-FEH* SSA. Red circles in panels (**C**,**D**) indicate haustoria. Bars = 3 mm. (**E**) RT-qPCR analysis of *Oc6-FEH* transcript levels in haustoria of plants transiently overexpressing *Oc6-FEH* via SSA, compared with EV. Data are means ± SD (*n* = 3). (**F**) FEH activity in haustoria of *Oc6-FEH*-overexpressing plants. Data are means ± SD (n = 3). (**G**) Fructan content in different tissues of the parasitic system: Root (far), host roots far from the parasite; Root (near), host roots adjacent to the parasite; haustoria; *O. cumana* stem tissues. Data are means ± SD (*n* = 3). Specific statistical tests used: Student’s *t*-test (**B**,**E**–**G**). * *p* < 0.05, *** *p* < 0.001; ns, not significant.

Recombinant *Oc6-FEH* was successfully expressed in *E. coli* as a GST fusion protein and confirmed by Western blot using a GST-specific antibody (Figure 3A), allowing for subsequent functional assays. The cell lysate supernatant was subjected to an in vitro fructan hydrolysis assay, and significant FEH activity was detected (Figure 3B).

Oc6-FEH was expressed in sunflower root by SSA transient overexpression and the seedling was infested by *O. cumana* (Figure 3C,D). RT-qPCR confirmed that the *Oc6-FEH* expression in the *Oc6-FEH* overexpression haustorium was 3.8-fold that in the control plant (Figure 3E). An enzyme assay indicated that the FEH activity in the *Oc6-FEH* overexpression haustorium was 1.8-fold that in the control plant (Figure 3F), indicating that *Oc6-FEH* has FEH activity in the plant. Compared with the control plant, the fructan content was decreased in all tested tissues in the *Oc6-FEH* overexpression plant. The fructan content was decreased by 27%, 65%, 58%, and 58% in the host far root, host near root, haustorium, and *O. cumana* tissues, respectively (Figure 3G), indicating that overexpression of *Oc6-FEH* specifically exacerbated fructan degradation at the parasitic interaction interface.

### 2.4. Effects of Oc6-FEH Overexpression on Oxidative Damage and Photosynthetic Activity Under Waterlogging Stress

**Figure 4:**
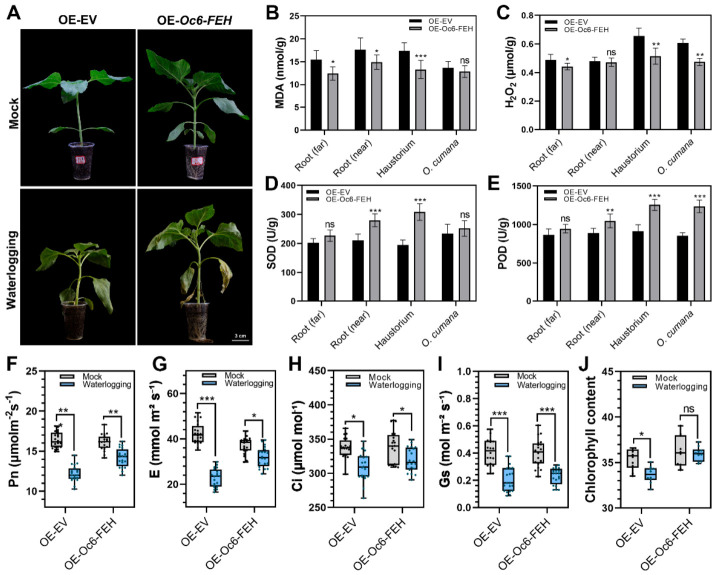
Overexpression of *Oc6-FEH* enhances antioxidant and photosynthetic activity in the sunflower–broomrape system. (**A**) Phenotype of pot-grown sunflower plants under control or waterlogging treatment. Plants transiently overexpressed empty vector (OE-EV) or *Oc6-FEH* (OE-Oc6-FEH). Bar = 3 cm. (**B**–**E**) Physiological indices related to oxidative stress in different tissues of the parasitic system after waterlogging treatment. (**B**) Malondialdehyde (MDA) content. (**C**) Hydrogen peroxide (H_2_O_2_) content. (**D**) Superoxide dismutase (SOD) activity. (**E**) Peroxidase (POD) activity. Tissues: Root (far), Root (near), haustoria, and *O. cumana.* Data are means ± SD (*n* = 10). (**F**–**I**) Photosynthetic parameters of host sunflower leaves after waterlogging treatment. (**F**) Net photosynthetic rate (Pn). (**G**) Transpiration rate (E). (**H**) Intercellular CO_2_ concentration (Ci). (**I**) Stomatal conductance (Gs). Data are means ± SD (*n* = 20). (**J**) Leaf chlorophyll content (SPAD value) of the host sunflower after waterlogging treatment. Data are means ± SD (*n* = 10). Specific statistical tests used: Student’s *t*-test (**B**–**J**). * *p* < 0.05, ** *p* < 0.01, *** *p* < 0.001; ns, not significant.

To determine whether overexpression of *Oc6-FEH* affects host performance under waterlogging stress, host sunflower plants overexpressing *Oc6-FEH* were subjected to waterlogging treatment. The control OE-EV sunflower showed typical stressed phenotypes such as dwarfing, wilting, and leaf yellowing under waterlogging stress. Overexpression of *Oc6-FEH* in the host sunflower significantly ameliorated these waterlogging-induced stress phenotypes (Figure 4A).

Under waterlogging stress, the accumulation of malondialdehyde (MDA) and hydrogen peroxide (H_2_O_2_) in all tested tissues of OE-*Oc6-FEH* plants was lower than that of the OE-EV control plant. Specifically, the MDA content decreased by 19.6%, 15.4%, and 23.7% in the host far root, host near root, and haustorium, respectively. The content of H_2_O_2_ decreased by 9.5%, 21.4%, and 21.8%, in the host far root, haustorium, and *O. cumana,* respectively (Figure 4B,C). The activities of two key ROS scavenging enzymes, superoxide dismutase (SOD) and peroxidase (POD), were significantly enhanced in most tissues of OE-*Oc6-FEH* plants compared with the OE-EV control plant. SOD activity increased by 33.0% and 58.0% in the host near root and haustorium, respectively. POD activity increased by 17.6%, 37.3%, and 44.8%, in the host near root, haustorium, and *O. cumana,* respectively (Figure 4D,E).

Photosynthesis is one of the core physiological processes suppressed by waterlogging, and the photosynthetic parameters are important indicators for this damage. Therefore, the photosynthetic parameters and chlorophyll content of the host leaves were measured. Under waterlogging stress, the net photosynthetic rate (Pn) and transpiration rate (E) of OE-EV control plants decreased significantly by 25.6% and 46.0%, respectively, while the decreases in OE-*Oc6-FEH* plants were only 11.8% and 14.0%, respectively, which were significantly milder than those in the OE-EV control plants (Figure 4F,G). Under waterlogging stress, the intercellular CO_2_ concentration (Ci) showed only minor changes in both groups, with an 8.2% decrease in OE-EV plants and a 5.4% decrease in OE-Oc6-FEH plants compared with their respective controls (Figure 4H). The stomatal conductance (Gs) significantly decreased in both groups under waterlogging stress, but the decrease in OE-*Oc6-FEH* plants (42.4%) was milder than that in the OE-EV control (48.7%) (Figure 4I). Waterlogging led to a 4.9% decrease in relative chlorophyll content (SPAD value) in the OE-EV plants, while the SPAD value was unchanged in OE-*Oc6-FEH* plants (Figure 4J).

### 2.5. Evaluation of Oc6-FEH in Waterlogging Tolerance of O. cumana

**Figure 5:**
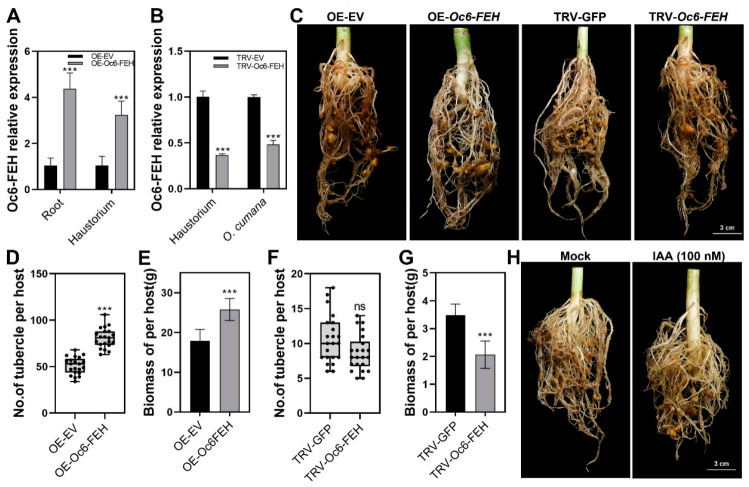
Functional validation of *Oc6-FEH* in waterlogging tolerance. (**A**) RT-qPCR analysis of *Oc6-FEH* transcript levels in the roots of host sunflower and in the haustoria of plants overexpressing OE-*Oc6-FEH* compared with OE-EV. Data are means ± SD (*n* = 3). (**B**) Silencing efficiency of *Oc6-FEH* via HIGS. RT-qPCR analysis of *Oc6-FEH* expression in haustoria and *O. cumana* stem tissues of plants infiltrated with TRV-*Oc6-FEH* compared with TRV-EV control. Data are means ± SD (*n* = 3). (**C**) Root phenotypes of sunflower plants under control or waterlogging treatment. Plants were transiently overexpressing empty vector (OE-EV), transiently overexpressing OE-*Oc6-FEH*, or subjected to HIGS with TRV-EV or TRV-*Oc6-FEH*. Bars = 3 cm. (**D**) Root phenotypes of sunflower plants treated with or without 100 nM IAA followed by waterlogging stress. (**D**,**E**) Effect of Oc6-FEH overexpression on parasitism by *O. cumana*. (**D**) Number of *O. cumana* tubercles per plant. (**E**) Total biomass of *O. cumana* per host plant. Data are means ± SD (*n* = 21). (**F**,**G**) Effect of *Oc6-FEH* silencing on parasitism by *O. cumana*. (**F**) Number of *O. cumana* tubercles per plant. (**G**) Total biomass of *O. cumana* per host plant. Data are means ± SD (*n* = 21). (**H**) Root phenotypes of sunflower plants treated with 100 nM IAA followed by waterlogging stress. Bar = 3 cm (**C**,**H**). Specific statistical tests used: Student’s *t*-test (**A**,**B**,**D**–**G**). *** *p* < 0.001; ns, not significant.

To directly verify the function of *Oc6-FEH* involved in waterlogging tolerance in parasitic plants, we utilized SSA and host-induced gene silencing (HIGS) techniques to achieve overexpression (OE) and silencing (TRV) of *Oc6-FEH* in parasitic plants. RT-qPCR validated the effectiveness of overexpression and interference, showing the upregulation and knockdown of *Oc6-FEH* expression in the root and haustorium by 4.4 and 3.2-fold and 63.4% and 51.7%, respectively (Figure 5A,B). After 3 days of waterlogging treatment on the above plants, the root phenotype is shown in Figure 5C. In control OE-EV plants, waterlogging caused browning and necrotic phenotypes in the attached *O. cumana*, while this phenotype was significantly ameliorated in OE-Oc6-FEH plants (Figure 5C). Further statistical analysis showed that overexpression of Oc6-FEH significantly promoted the parasitic rate of *O.cumana*, with a 58.0% increase in parasitism and a 44.4% increase in biomass compared with the OE-EV control under waterlogging stress (Figure 5D,E). On the contrary, the browning and necrotic phenotype of TRV-*Oc6-FEH* plants was more severe than that of control TRV-EV plants (Figure 3C). Furthermore, the TRV interference of *Oc6-FEH* decreased the parasite biomass by 40.7% compared with control TRV-EV plants under waterlogging stress, while it did not influence the tubercle number (Figure 5F,G).

Following 3 days of waterlogging, IAA treatment effectively reduced the browning and necrotic phenotypes in both sunflower roots and attached *O. cumana* tubercles (Figure 5H).

## 3. Discussion

We focused on the expression pattern, enzymatic function, and physiological role of a fructan exohydrolase gene, *Oc6-FEH*, in a parasitic plant system under waterlogging stress. The expression of *Oc6-FEH* was significantly upregulated by flooding, exogenous fructan (hydroponic system), IAA, and MeJA. The encoded protein exhibited fructan 6-exohydrolase activity. Overexpression of *Oc6-FEH* in sunflower enhanced waterlogging tolerance by mitigating oxidative stress and maintaining photosynthetic performance at higher levels. Furthermore, the expression level of *Oc6-FEH* was positively correlated with both host waterlogging tolerance and the parasitic success rate. Together, these findings demonstrate that *Oc6-FEH* contributes to plant adaptation to waterlogging and plays a critical role in parasitic interaction.

In the plant genome, FEHs are evolutionarily closely related to cell wall invertases (CWINVs), both belonging to glycoside hydrolase family 32 (GH32) [41]. Oc6-FEH is likely to originate from cell wall convertase and to acquire the ability to specifically hydrolyze fructan through gene replication and functional differentiation events [42,43]. In this study, *Oc6-FEH* was cloned from the obligate root parasite *O. cumana*, a plant that has completely lost photosynthetic capacity. Under such extreme nutritional specialization, gene families involved in carbohydrate metabolism, transport, and sensing are expected to have undergone strong natural selection [44,45]. We hypothesize that, in broomrape, *Oc6-FEH* may have retained some ancestral functions related to cell wall metabolism, potentially participating in processes such as carbon source perception or cell wall modification during parasitism. Additionally, it may have evolved to perceive and respond to specific fructan-derived signals from the host or environment.

Waterlogging typically induces root hypoxia in plants, triggering comprehensive metabolic reprogramming that includes the accumulation of soluble sugars [46,47,48]. We speculate that the endogenous fructan metabolism mediated by *Oc6-FEH* could be a key initiator of such adaptive metabolic shifts. In supporting a role for fructan turnover in stress sensing, expression of the maize *Zm-6-FEH* gene is strongly induced by exogenous fructan oligosaccharides [32]. Fructans serve as a major reserve of soluble carbohydrates, and their hydrolysis can rapidly release fructose and small amounts of glucose to meet energy and osmotic demands [32,49]. Therefore, the mobilization of fructan reserves by *Oc6-FEH* may contribute to maintaining cellular energy status and osmotic balance under waterlogged conditions. FEH-mediated dynamic remodeling of carbon reserves may represent a conserved adaptive strategy in plants under adverse conditions.

In this study, we found that *Oc6-FEH* expression is rapidly induced by waterlogging stress. Promoter analysis revealed that its 2000 bp upstream region contains putative anaerobic-responsive elements, as well as IAA and MeJA-response motifs (Figure 2A). Consistent with this, exogenous application of IAA and MeJA strongly upregulated *Oc6-FEH* expression. Previous studies have established that waterlogging triggers ethylene biosynthesis, which subsequently influences IAA synthesis, transport, and signaling to regulate aerenchyma formation and adventitious root development [50,51,52]. In our experiments, IAA treatment not only induced *Oc6-FEH* expression but also alleviated waterlogging-induced root browning and necrosis. Given that IAA induces Oc6-FEH expression (Figure 2) and exogenous IAA application mitigates waterlogging-induced damage to the parasitic system (Figure 5H), we propose that IAA enhances waterlogging tolerance at least partly through the upregulation of Oc6-FEH.

Waterlogging triggers the overproduction of ROS and severely compromises photosynthetic capacity [53]. Overexpression of *Oc6-FEH* in the host plant alleviates waterlogging damage, an effect correlated with the modulation of antioxidant defenses and photosynthetic performance. Plants transiently overexpressing *Oc6-FEH* exhibited significantly reduced accumulation of MDA and H_2_O_2_, alongside enhanced activities of antioxidant enzymes such as SOD and POD. This suggests that *Oc6-FEH* bolsters the antioxidant capacity of the parasitic system, likely through its fructan hydrolase activity. First, the monosaccharides released from fructan degradation can directly enter metabolic pathways like the pentose phosphate pathway to generate NADPH, thereby supplying the reducing power required for antioxidant cycles [54]. Second, fructans or their breakdown products may act as signaling molecules that indirectly upregulate the expression of antioxidant enzyme genes (e.g., SOD and POD) by modulating sugar-sensing pathways. Under waterlogging, OE-Oc6-FEH plants maintained better photosynthetic performance than OE-EV controls, as indicated by a milder decline in photosynthetic parameters (Figure 4). The preservation of photosynthetic function is critical for sustaining carbon assimilation and the overall survival of the parasitic system under waterlogging stress [55,56,57]. We speculate that *Oc6-FEH* protects the integrity of the photosynthetic system by mitigating oxidative damage. The soluble sugars released through fructan degradation may also contribute to osmotic adjustment [58]. Although we observed reduced fructan content upon *Oc6-FEH* overexpression, we did not directly measure fructose levels. Future studies could examine fructose accumulation to further confirm the catalytic activity of Oc6-FEH in vivo. It has been proposed that fructans and their breakdown products can themselves act as signaling molecules [49]. Therefore, *Oc6-FEH* likely functions not only as a metabolic enzyme but also as a critical component in coupling stress perception with downstream physiological adaptations.

*Oc6-FEH* not only influences abiotic stress tolerance but also acts as a positive regulator for the successful parasitism of *O. cumana*. Overexpression of *Oc6-FEH* significantly increased the number of tubercles and the biomass of the parasite under waterlogged conditions, whereas silencing its expression had the opposite effect (Figure 5D–G). This indicates that *Oc6-FEH* function is closely linked to the parasitic establishment process. Interestingly, although OE-*Oc6-FEH* plants supported a higher number of parasites under waterlogging stress (Figure 5D), they simultaneously exhibited improved oxidative status and photosynthetic performance compared with OE-EV controls (Figure 4). This seemingly paradoxical observation suggests that *Oc6-FEH* enhances host waterlogging tolerance to an extent that compensates for the increased parasitic burden. In other words, the direct effect of *Oc6-FEH* on host stress adaptation appears to outweigh any potential negative consequences of supporting more parasites. These findings highlight the multifaceted role of *Oc6-FEH* in the parasitic system: it not only benefits the parasite by promoting its establishment under stress, but also confers enhanced stress tolerance to the host, ultimately improving the overall performance of the sunflower–broomrape system under waterlogging stress. Successful host invasion by parasitic plants depends on the proper development and function of the haustorium, a process—including its initiation, host penetration, and vascular connection—that is tightly regulated by developmental signals [44,59]. Fructan metabolism may be associated with plant development. In some species, FEH activity correlates with processes such as cell division and vascular differentiation [25]. Although the specific role of *6-FEH* in development is not fully defined, its homologs, such as CWINs, are well-established regulators of source–sink relationships, organ size, and vascular development [60]. CWINs hydrolyze sucrose to supply hexoses to rapidly growing tissues and elicit broad signaling effects [60]. We speculate that *Oc6-FEH* may influence parasitism through a functionally analogous pathway.

In this study, all functional analyses were conducted in the sunflower–broomrape system, where waterlogging and parasitism stresses coexist—a scenario that reflects natural conditions. While our results demonstrate that *Oc6-FEH* overexpression improves host performance under these combined stresses, the experimental design does not fully separate the direct effects on host waterlogging tolerance from potential indirect effects mediated by altered parasitic burden. However, the observation that OE-*Oc6-FEH* plants performed better despite harboring more parasites (Figure 5D) strongly suggests that *Oc6-FEH* enhances host stress tolerance directly. Future studies incorporating non-parasitized controls will help further quantify the relative contributions of host tolerance and parasitic load to the overall phenotype.

## 4. Materials and Methods

### 4.1. Plant Material

Sunflower cultivar SH361 was provided by the Inner Mongolia Academy of Agriculture and Animal Husbandry Sciences. *O. cumana* race G seeds were collected from a naturally infested field in Hohhot, China (41.09 N, longitude, 111.46 E) during autumn 2022. The seeds were surface-sterilized by sequential treatment with 2% (*v*/*v*) sodium hypochlorite for 10 min and 70% (*v*/*v*) ethanol for 5 min, followed by five rinses with sterile distilled water, then dried on sterile filter paper. No specific permits were required for the collection of *O. cumana* seeds, as this species is a common agricultural weed in the region and the collection site was not protected. This study complies with institutional, national, and international guidelines for plant research.

### 4.2. Hydroponic System and O. cumana Inoculation

A hydroponic system was established for root culture and parasitic experiments. Square Petri dishes (12 cm × 12 cm) were lined with a layer of rock wool as a supporting substrate, overlaid with two layers of sterile filter paper moistened with ddH_2_O. Sunflower seedlings were transplanted onto the filter paper and cultured in a growth chamber (Yanyi Scientific Instrument Co., Ltd., Shanghai, China) under the following conditions: temperature 25 °C ± 2 °C, photoperiod 16 h light/8 h dark, relative humidity 60–70%, and light intensity approximately 8000 Lux for 5 days to allow for root system adaptation, maintaining the filter paper moist but not waterlogged.

For *O. cumana* inoculation, 0.02 g of surface-sterilized seeds (sterilized as described in Section 4.1) were evenly sprinkled onto the rhizosphere of the sunflower seedlings. At 24 h post-inoculation, 1 mL of 100 nM rac-GR24 (a synthetic strigolactone analog) was applied to the roots of each seedling to stimulate *O. cumana* seed germination. The seedlings were further cultured under the same conditions for 15 days (temperature 25 °C ± 2 °C, photoperiod 16 h light/8 h dark, relative humidity 60–70%, and light intensity approximately 20,000 Lux).

### 4.3. Soil Culture System for O. cumana Inoculation and Evaluation of Parasitism

For soil culture experiments, *O. cumana* seeds (20 mg) were thoroughly mixed with a sterilized substrate composed of vermiculite and soil at a 1:1 (*v*/*v*) ratio (100 mL vermiculite and 100 mL soil). The seed–substrate mixture was placed into plastic pots (300 mL volume) and covered with a thin layer (approximately 1 cm) of the same substrate mixture to ensure a uniform distribution of parasite seeds in the sunflower root zone.

Sunflower seedlings were initially grown in germination trays under controlled conditions (25 °C, 16 h light/8 h photoperiod) for 10 days until they reached the two-true-leaf stage. Uniformly developed seedlings were selected and carefully transplanted into the prepared pots containing the *O. cumana* seed–substrate mixture. Each pot contained one sunflower seedling. The pots were then placed in a greenhouse under controlled conditions: temperature 25 °C ± 2 °C, photoperiod 16 h light/8 h dark, relative humidity 60–70%, and light intensity approximately 20,000 Lux. Plants were watered regularly to maintain adequate soil moisture without waterlogging.

For waterlogging treatment, uniformly grown plants were selected. The pots were placed in a large container filled with water, and the water level was maintained at approximately 2 cm above the soil surface to ensure continuous waterlogging stress on the roots. Control plants were watered normally to keep the soil moist but not waterlogged. Thirty plants were randomly assigned to a control or waterlogging group (15 each), and three plants per group were sampled at 0, 3, 6, 9, and 12 days.

At 45 days post-inoculation, sunflower roots were carefully removed from the pots and gently washed with tap water to remove soil and vermiculite particles. The number of *O. cumana* tubercles attached to the sunflower root system was recorded. The fresh weight of *O. cumana* tubercles per host plant was recorded immediately after counting.

### 4.4. Hormone and Chemical Treatments

All plant hormone working solutions were prepared fresh prior to use. Indole-3-acetic acid (IAA) was first dissolved in a small volume of ethanol, and methyl jasmonate (MeJA) was directly dissolved in deionized water containing 0.1% (*v*/*v*) Tween-20 (Beijing Solarbio Science & Technology Co., Ltd., Beijing, China). All working solutions were diluted to their final concentrations using deionized water supplemented with 0.1% Tween-20, and this solution served as the solvent control. The levan-type fructan used in the experiments was directly dissolved in deionized water, with deionized water used as the control.

For the promoter activity assay, 4-week-old *Nicotiana benthamiana* plants with 5–6 fully expanded leaves were used. The leaves were sprayed with working solutions of either 100 nM IAA or 100 μM MeJA until the leaf surfaces were fully wet. Tissues were harvested 24 h after treatment for GUS histochemical staining and RT-qPCR analysis. To verify the inductive effect of fructan on Oc6-FEH, 25-day-old sunflower plants were sprayed with a 0.02% (*w*/*v*) fructan aqueous solution. The treatment was repeated every 48 h for a total duration of 7 days before parasitic tissues were collected for expression analysis. For the exogenous treatment in the parasitic system and waterlogging tolerance phenotyping assay, the treatment method was modified to the addition of 1 mL of 100 mM IAA working solution directly into the plant container. Twenty-four hours after the addition, plants were subjected to waterlogging stress for 3 days, after which root phenotypes were evaluated and parasitic parameters were quantified.

### 4.5. Gene Expression Analysis

Total RNA was extracted using Trizol reagent (TransGen Biotech Co., Ltd., Beijing, China) according to the manufacturer’s protocol. Potential genomic DNA contamination was eliminated by DNase I treatment. RNA quality and concentration were assessed with a NanoDrop2000 spectrophotometer (Thermo Fisher Scientific, Waltham, MA, USA). First-strand cDNA was synthesized from equal amounts of total RNA using the HiScript III RT SuperMix for qPCR kit (Vazyme Biotech Co., Ltd., Nanjing, China). For qPCR analysis, specific primers were designed, and *HaActin* (Accession No. AF282624) and *OcActin* (Unigene ID: Cluster-9748.42356) were used as internal reference genes for sunflower and *O. cumana*, respectively. Quantitative PCR was performed on a qTOWER2.2 real-time PCR system (Analytik Jena GmbH, Jena, Germany) using TransStart Tip Green qPCR SuperMix (TransGen Biotech Co., Ltd., Beijing, China). The amplification program was 95 °C for 2 min, followed by 40 cycles of 95 °C for 15 s and 60 °C for 30 s, with a melting curve analysis to verify product specificity. Relative expression levels were calculated using the 2^−ΔΔCt^ method. Each reaction was run with three technical replicates, and the data were obtained from three independent biological replicates.

### 4.6. Host-Induced Gene Silencing (HIGS) Vector Construction and Transformation

To achieve HIGS against *O. cumana*, we employed a TRV-based Virus-Induced Gene Silencing (VIGS) system. Vectors for TRV-based VIGS, including pTRV1, pTRV2 (pYL279), and the control vector pYL36 (TRV-GFP), were kindly provided by Dr. Dinesh Kumar [61,62]. Gene-specific sequences were retrieved from the sunflower transcriptome database. To ensure targeting specificity, a 300–400 bp fragment with minimal homology to other genes (containing a unique stretch of at least 17 nt) was selected for each target and cloned into the pTRV2 backbone. All recombinant vectors were separately introduced into *Agrobacterium tumefaciens* strain GV3101 by electroporation.

For agroinfiltration, *A. tumefaciens* cultures carrying pTRV1 or the respective pTRV2 constructs were grown to mid-log phase (OD_600_ = 1.6). Bacterial cells were collected by centrifugation and resuspended in induction buffer containing 10 mM MES (pH 5.6), 10 mM MgCl_2_, and 200 µM acetosyringone. Immediately before the Seed Soak Agroinfiltration (SSA) treatment, the pTRV1 suspension was mixed with each pTRV2 suspension at a 1:1 (*v*/*v*) ratio to prepare the final inoculum.

### 4.7. SSA Assay

The SSA assay was carried out following an established and validated protocol for transient overexpression in sunflower [63]. Briefly, sunflower seeds were manually debusked to remove both the outer and inner seed coats. The exposed seeds were surface-sterilized by immersion in 75% (*v*/*v*) ethanol for 3–5 min, followed by five washes with sterile distilled water. To enable *Agrobacterium* entry, the seed surface was uniformly scarified by making gentle, superficial incisions with a sterile inoculation needle. The scarified seeds were then immersed in the prepared *Agrobacterium* inoculum inside a vacuum desiccator. A vacuum was slowly applied to –0.05 to –0.08 MPa, maintained for 2 min, and then gradually released. This vacuum-infiltration step was repeated once. After vacuum treatment, seeds were kept in the bacterial suspension for an additional 5 h in darkness. Seeds were then rinsed thoroughly with sterile water, blotted dry on sterile filter paper, placed on two layers of moistened filter paper in a tray, and covered with two additional moist filter papers to maintain humidity. Seeds were incubated in a growth chamber under the following conditions: 25/21 °C (day/night, ±1 °C), 16 h light/8 h dark photoperiod, light intensity of 200 μmol m^−2^ s^−1^, and 60–70% relative humidity. After 10 days, seedlings were transplanted into soil for further cultivation.

### 4.8. Fructan Content Determination

Fructan accumulation in plant tissues was examined using a commercial enzyme-linked immunosorbent assay kit (Cat# MM-6353001, Enzyme-linked Biotechnology, Shanghai, China). Frozen samples were homogenized in ice-cold PBS (0.1 M, pH 7.4) and centrifuged at 12,000 *g* for 10 min at 4 °C. The resulting supernatant was diluted 1:5 with PBS and loaded onto microplates pre-coated with fructan-capture antibody. Following incubation at 37 °C for 30 min, plates were washed five times with PBST (0.05% Tween-20 in PBS). HRP-conjugated detection antibody was then applied and incubated for another 30 min at 37 °C. After thorough washing, TMB substrate was added and the plates were kept in the dark at 37 °C for 15 min. The chromogenic reaction was stopped with 2 M H_2_SO_4_, and absorbance was immediately read at 450 nm. A standard curve spanning 0–40 ng/mL was generated using purified fructan as the standard, and fructan content was quantified accordingly. Fructan content was normalized to tissue fresh weight and is expressed as ng g^−1^ FW. Three biological replicates were included for each measurement.

### 4.9. Photosynthetic Parameter Measurement

Gas exchange parameters of sunflower leaves were measured using a portable photosynthesis system (LI-6800, LI-COR Biosciences, Lincoln, NE, USA) equipped with a standard leaf chamber (6800-01A fluorometer chamber). Measurements were conducted on the youngest fully expanded leaves between 9:00 AM and 11:30 AM to ensure optimal physiological activity. During measurement, the conditions within the leaf chamber were controlled as follows: the photosynthetically active photon flux density (PPFD) was set at 2000 μmol m^−2^ s^−1^ and provided by the integrated LED light source; the CO_2_ concentration in the sample cell was controlled at 400 μmol mol^−1^ using a CO_2_ mixer; the leaf temperature was maintained at 25 °C; and the relative humidity was controlled at 60%. The flow rate of air through the system was maintained at 600 μmol s^−1^, with the pump set to “Auto” mode. The chamber mixing fan speed was set to 10,000 rpm to ensure adequate boundary layer conductance. The leaf was placed in the chamber and allowed to acclimate under the set conditions until the readings stabilized (coefficient of variation < 1%) before data were logged.

Additionally, the relative chlorophyll content (SPAD value) of the same leaves was measured immediately after gas exchange recordings using a portable chlorophyll meter (SPAD-502, Konica Minolta, Tokyo, Japan).

### 4.10. Determination of ROS-Related Parameters

MDA content was measured using the thiobarbituric acid (TBA) method. Fresh tissue samples (approximately 0.1 g) were homogenized in 1 mL of ice-cold extraction buffer and centrifuged at 12,000 *g* for 10 min at 4 °C. The supernatant was collected and mixed with TBA reaction solution. The mixture was incubated in a boiling water bath for 30 min, immediately cooled on ice, and centrifuged at 12,000 *g* for 10 min. The absorbance of the supernatant was read at 532 nm and 600 nm. MDA content was calculated using the following formula: MDA content (nmol g^−1^ FW) = 32.3 × ΔA ÷ W, where ΔA = A_532_ − A_600_ and W is the fresh weight (g).

H_2_O_2_ content was determined by a chromogenic method. Fresh tissue samples (approximately 0.1 g) were homogenized in 1 mL of ice-cold extraction buffer and centrifuged at 12,000 *g* for 10 min at 4 °C. The supernatant was incubated with a specific chromogenic reagent for 5 min at room temperature, and the absorbance was measured at 510 nm. H_2_O_2_ content was calculated based on a standard curve (y = 14.429x − 0.0115, where x is the H_2_O_2_ amount in μmol and y is ΔA) and is expressed as μmol g^−1^ FW. When necessary, samples were diluted, and the dilution factor was applied to the calculation.

### 4.11. Determination of SOD, POD, and FEH Activities

Superoxide dismutase (SOD) activity was assayed using the WST-8 method. Fresh tissue samples (approximately 0.1 g) were homogenized in ice-cold extraction buffer and centrifuged at 12,000 *g* for 10 min at 4 °C. The supernatant was collected and subjected to the WST-8 reaction system. Absorbance was recorded at 450 nm. One unit (U) of SOD activity was defined as the amount of enzyme required to cause 50% inhibition of the reaction under the assay conditions. SOD activity is expressed as U g^−1^.

Peroxidase (POD) activity was measured via the guaiacol oxidation method. The supernatant was mixed with a reaction mixture containing guaiacol and H_2_O_2_, and the increase in absorbance at 470 nm was immediately monitored for 1 min. One unit (U) of POD activity was defined as the amount of enzyme that caused an increase of 1 in absorbance at 470 nm per minute per gram fresh weight. POD activity is expressed as U g^−1^.

Fructan exohydrolase (FEH) activity was determined using the 3,5-dinitrosalicylic acid (DNS) method. For plant tissues, samples were homogenized in ice-cold 95% ethanol, sequentially washed with ethanol and extraction buffer, and the final supernatant was collected as crude enzyme extract. For recombinant protein, *E. coli* cells expressing Oc6-FEH were harvested, lysed by sonication, and the supernatant was recovered after centrifugation. The enzyme extract was incubated with fructan substrate at 95 °C for 10 min, and the absorbance of the reaction product was measured at 540 nm. One unit of FEH activity was defined as the amount of enzyme that produced 1 μg of reducing sugars per hour. FEH activity was normalized to fresh weight for plant tissues or to protein content for recombinant samples. All measurements were performed with three independent biological replicates.

### 4.12. Statistical Analysis

Data were analyzed using SPSS software (version 25.0, IBM Corp., Armonk, NY, USA). Normality and homogeneity of variances were verified using Shapiro–Wilk and Levene’s tests, respectively. For data meeting parametric assumptions, one-way ANOVA followed by the Least Significant Difference (LSD) test was used for multiple comparisons, and Student’s *t*-test was used for two-group comparisons. Significance levels are indicated as * p < 0.05, ** p < 0.01, and *** p < 0.001; ns, not significant, with different lowercase letters denoting significant differences in post-hoc tests. All data are presented as means ± SD from at least three independent biological replicates.

## 5. Conclusions

This study demonstrates that *Oc6-FEH*, a fructan 6-exohydrolase from the parasitic plant *O. cumana*, plays a dual role in waterlogging tolerance. *Oc6-FEH* expression is induced by waterlogging stress, IAA, and MeJA, and its overexpression in host sunflower alleviates oxidative damage, maintains photosynthetic performance, and enhances waterlogging tolerance. Furthermore, *Oc6-FEH* promotes successful parasitism of *O. cumana* under waterlogged conditions. These findings reveal a novel mechanism by which fructan metabolism contributes to stress adaptation in parasitic plant systems and identify *Oc6-FEH* as a promising genetic target for improving flooding tolerance in crops.

## Data Availability

The *Oc6-FEH* sequence was retrieved from the *O. cumana* transcriptome data (BioProject PRJNA635007). Other gene sequences were obtained from the NCBI database. All other data supporting the findings of this study are available within the article. Raw data are available from the corresponding authors upon reasonable request.

## Supplementary Materials

The following supporting information can be downloaded at: https://www.mdpi.com/article/10.3390/plants15091326/s1, Figure S1. Multiple sequence alignment of Oc6-FEH from *Orobanche cumana* with 6-FEH proteins from *Arabidopsis thaliana* (At6-FEH) and *Beta vulgaris* (Bv6-FEH). Three conserved motifs (NDPNG, FRD, and WEC) that are characteristic of GH32 family 6-FEHs are highlighted by red boxes. Table S1. Gene primer information.
