# Supplementary material for: A Fructan 6-Exohydrolase from Orobanche cumana Boosts Waterlogging Tolerance in Parasitic and Root Tissues
Source: Plants (Basel). 2026 Apr 27;15(9):1326. doi: 10.3390/plants15091326 (PMC13164756; doi:10.3390/plants15091326)
Supplement: Supplementary file 1 [file plants-15-01326-s001.zip › Supplementary Figure S1.pdf]

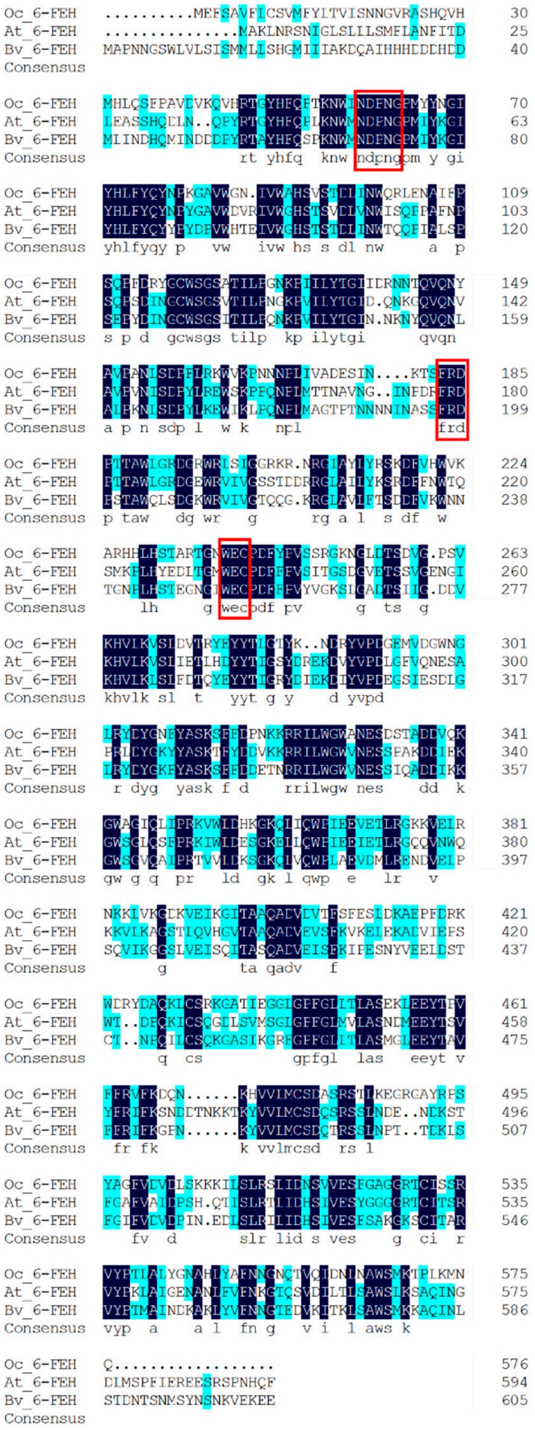

Fig. S1. Multiple sequence alignment of Oc6-FEH from *Orobanche cumana* with 6-FEH proteins from *Arabidopsis thaliana* (At6-FEH) and *Beta vulgaris* (Bv6-FEH). Three conserved motifs (NDPNG, FRD, and WEC) that are characteristic of GH32 family 6-FEHs are highlighted by red boxes.
